# Acantholytic oral squamous cell carcinoma with clear cell change - a rare amalgamated variant

**DOI:** 10.4322/acr.2023.450

**Published:** 2023-10-11

**Authors:** Gitika Sharma, Anju Devi, Mala Kamboj, Anjali Narwal

**Affiliations:** 1 Pandit Bhagwat Dayal Sharma University of Health Sciences, Oral Pathology and Microbiology, Rohtak, Haryana, India

**Keywords:** Oral Squamous Cell Carcinoma, Acantholytic Squamous Cell Carcinoma, Clear Cells, immunohistochemistry

## Abstract

**Background:**

Acantholytic squamous cell carcinoma (ASCC) is an uncommon histological variation of oral squamous cell carcinoma (OSCC), accounting for fewer than 4% of all occurrences. The tumor shows a slight masculine predisposition, with the lower lip being the most commonly affected location. ASCC is reported to have a diverse biologic behavior, which explains its ability to metastasize to distant places and, thus, its poor prognosis. Similarly, clear cell change in OSCC is a rare occurrence with an unknown etiology that suggests its aggressive nature.

**Method and Results:**

Histopathology reveals central acantholytic cells with numerous duct-like features. The presence of distinct cytological atypia contributes to the diagnosis of SCC. Special stains and IHC aid in distinguishing tumor from other histopathologically similar entities.

**Conclusion:**

The case of a 29-year-old male presented here with an updated literature review highlights the need for histological study of the unique and seldom seen oral ASCC with clear cell change, which can be ignored because of similarities with other entities. Because recurrence rates are so high for ASCC, amalgamated clear cell change makes it critical for proper treatment initiation with a definite diagnosis. To the best of our knowledge, this is the first documented occurrence. Our experience with the present case suspected a more aggressive behavior due to a high Ki-67 index, anticipating a poorer prognosis in the oral cavity considering the patient's young age.

## INTRODUCTION

Oral squamous cell carcinoma (OSCC) is the eleventh most common cancer worldwide, accounting for 3% of all malignancies and 92-95% of all mouth cancers.^1^ Estimated 300,000 new cases and 145,000 deaths in 2012, as well as 702,000 prevalent cases over five years, establish it as a worldwide health problem.^2^ Conventional OSCC can manifest as numerous forms that account for approximately 10-15% of all squamous cell carcinomas (SCC). Basaloid SCC, acantholytic SCC, adenosquamous carcinoma, spindle cell/sarcomatoid carcinoma, papillary SCC, and lymphoepithelial carcinoma are the histological subtypes classified by the World Health Organization (WHO) in 2022.^3^ Amidst these, basaloid and acantholytic SCC have a significant impact on biological behavior and prognosis. Thus, distinguishing these histological subtypes from ordinary OSCCs is critical.^4^ Acantholytic squamous cell carcinoma is an unusual, histologically distinct type of SCC that was initially described by Lever in 1947 and affects sun-exposed skin.^5^ ASCC has a cumulative incidence of 0.1%.^6^ It’s exceptional rarity in the oral cavity distinguishes it as a standalone histopathological variety. The peak of oral ASCC occurs in the sixth or seventh decade.^7^ Further, OSCC with prominent clear-cell differentiation is uncommon with unknown etiology. Kuo^8^ discovered it in the skin in 1980 and named it hydropic SCC. Clear-cell change can be detected in any neoplasms, but it is uncommon to locate as a pure-form variation in head-and-neck SCC. Only nine cases of typical OSCC with obvious cell change in the oral cavity have been documented. Despite literature detailing its prevalence and biological behavior, the WHO has yet to recognize the clear cell variation in classifying oral cavity tumors due to its great rarity.^9^ Several authors proposed that obvious cell change could signify an advanced stage of OSCC, implying an aggressive nature with a bad prognosis.^10^ We present the case of a 29-year-old male with oral ASCC with additional clear cell change. This case report aims to highlight the appearance of an exceptionally unusual example of oral ASCC with evident cell change and its relationship with the tumor's biological behavior.

## CASE REPORT

**Figure 1:**
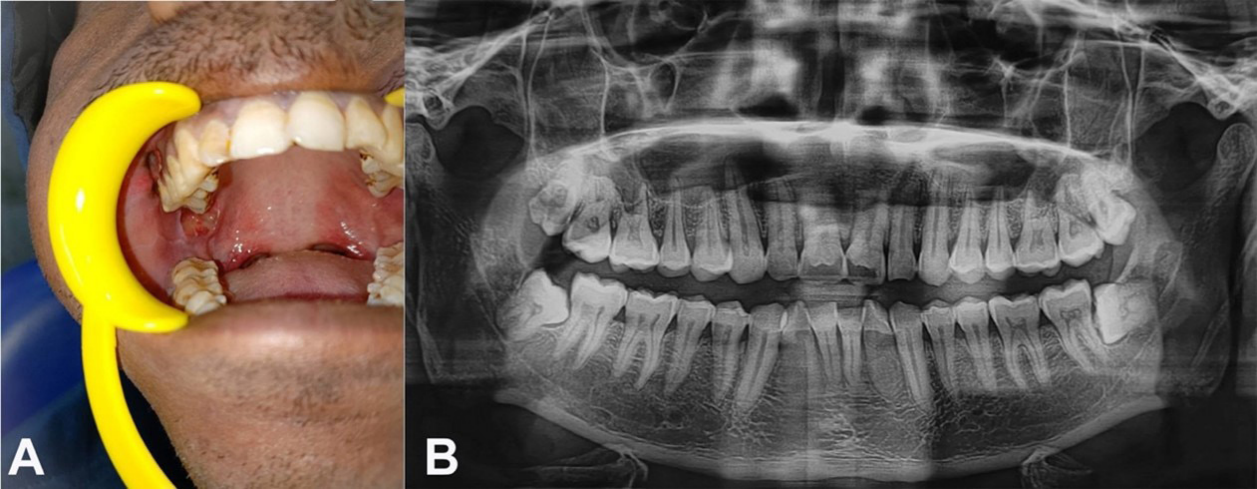
**A -** intraoral photograph revealing ulceroproliferative lesion on right pterygomandibular raphe; **B -** orthopantogram without soft tissue or bony changes.

A 29-year-old male sought health care with a painful ulcer on the right inner cheek region that had been present for one month. The patient was apparently normal for a month before seeing an ulcer on the inner side of the cheek’s region. Ulcers were painful and exacerbated by spicy foods. He claimed to be an avid hukkah smoker twice a day for the past five years. One and a half years ago, the patient was treated for tuberculosis and got treatment for it. An ulceroproliferative lesion was seen intraorally on the right pterygomandibular raphe, which was indurated, tender on palpation, and covered with necrotic slough (Figure 1A). On radiographic examination, orthopantomogram detected no soft tissue or bony changes (Figure 1B).

**Figure 2:**
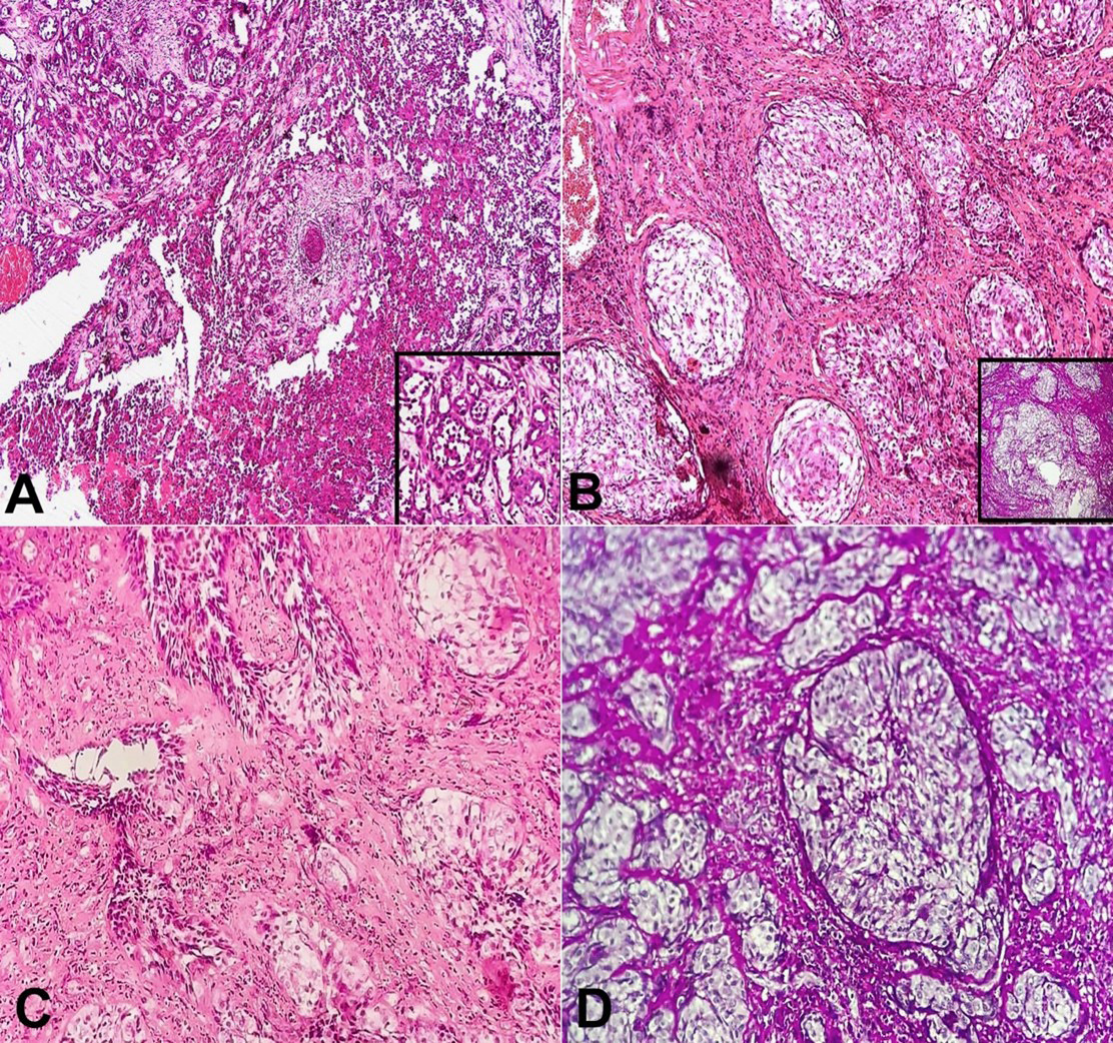
Photomicrographs of the biopsy. **A -** photomicrograph reveals dysplastic islands showing acantholytic cells (H&E, 20X); **B -** dysplastic islands showing clear cell change (H&E, 20X); **C -** acantholytic and clear cell change (H&E, 20X); **D -** tumor showing PAS-negative pseudoglandular appearance (20X).

Based on clinical-radiographic correlation, a provisional diagnosis of a malignant ulcer was raised. Clinically, the differential diagnosis of tuberculous ulcer was given. Exfoliative cytology revealed clusters of cells with altered nucleo-cytoplasmic ratio and patient was advised a biopsy. The acid-fast bacillus stain was negative, ruling out a differential diagnosis of tuberculosis. An incisional biopsy was performed under local anesthesia, suggesting ASCC with clear cell change. The patient underwent surgical resection, and the specimen was sent to the Department of Oral Pathology. The invasion of atypical epithelial cells into the connective tissue in the form of islands, strands, and cords was revealed by microscopic analysis of the H&E-stained section. These islands exhibited atypical characteristics such as cellular and nuclear pleomorphism, an elevated nuclear/cytoplasmic ratio with conspicuous nucleoli, and many mitotic figures. The atypical islands’ center had dyscohesive cells with hyperchromatic nuclei and eosinophilic cytoplasm (Figure 2A). Within these islands, there was also clear cell change (Figure 2B, 2C). A single layer of flat to polygonal cells with central acantholysis bordered the duct-like or pseudo-glandular structures. The PAS stain- indicated that these formations were non-glandular and lacked mucinous components (Figure 2D). There was no evidence of keratin pearl development across the tumor bulk. The stroma of the surrounding connective tissue was fibro-cellular, with persistent inflammatory cell infiltration.

**Figure 3:**
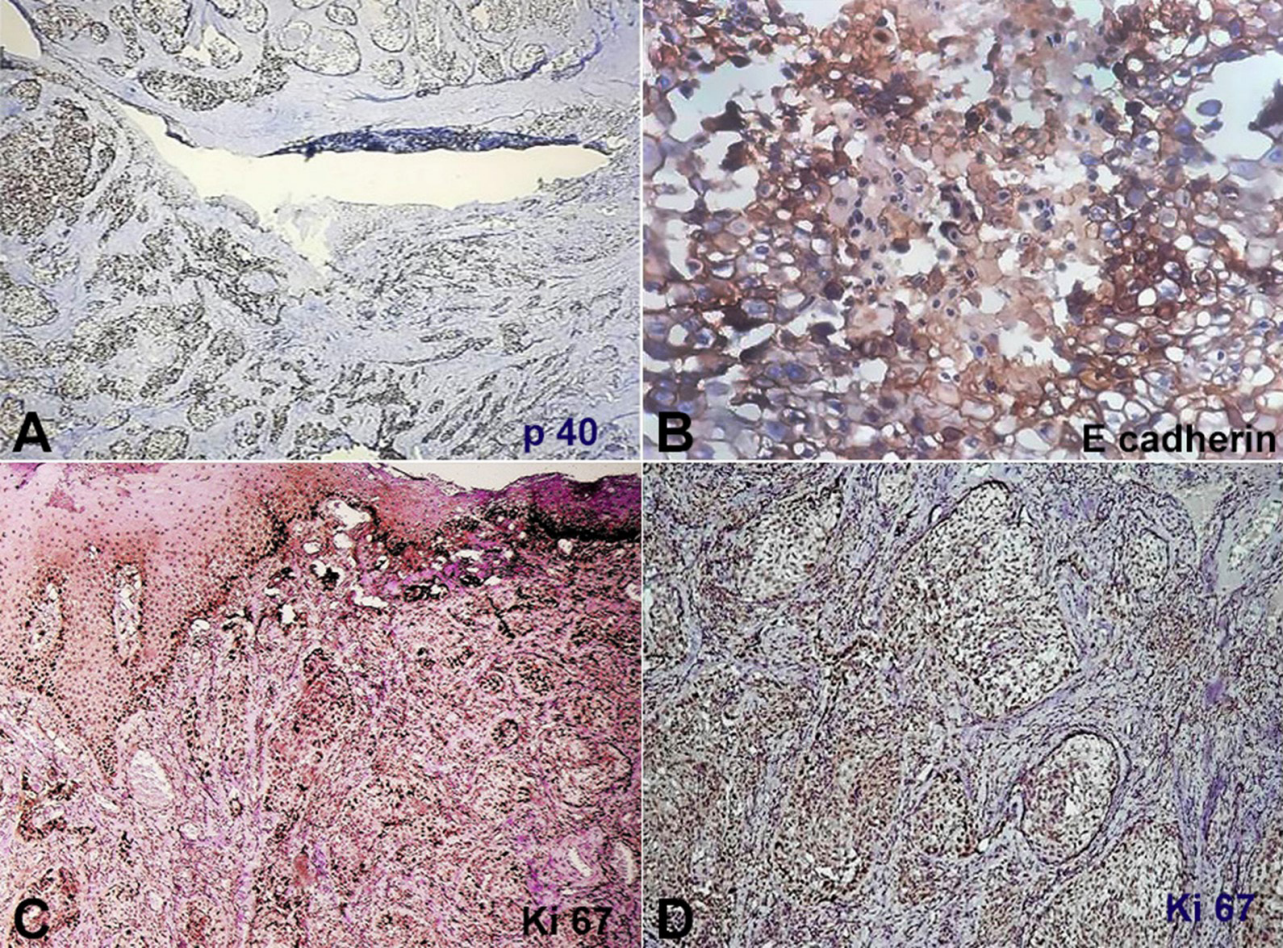
Photomicrographs of the biopsy. Immunohistochemical reactions results for Acantholytic squamous cell carcinoma. **A -** positive reaction for p40 (10X); **B -** weak reaction for E-cadherin in acantholytic cells (20X); **C** and **D -** high index of Ki67 (4X and 20X respectively).

There was also muscle invasion. These neoplastic acantholytic cells were immunohistochemically (IHC) revealed to be diffusely positive for p40. The acantholytic cells within the tumor islands showed a robust expression of p40, a high Ki-67 index, and loss of E-cadherin (Figure 3A, 3B, 3C and 3D). After considering all the histological findings and IHC results, the final diagnosis of acantholytic SCC with clear cell change was made.

## DISCUSSION

The keywords 'Acantholytic,' 'Oral,' 'Clear cell,' and 'SCC' were entered into the Pubmed database. Non-head and neck tumors are not included. The cases were retrieved and tabulated with extensive clinicopathological data (Table 1).

**Table 1:** Table showing details of previously documented cases of Acantholytic and clear cell SCC

**Ref.**	**Age/Sex**	**Location**	**Clinical Features**	**H/P diagnosis**	**Recurrence**	**Follow up**
^7^	58/ F	Gingiva (mandible)	Exophytic mass	ASCC	N/A	N/A
	57/M	Tongue	N/A	ASCC	N/A	N/A
	68/M	Tongue	N/A	ASCC	N/A	N/A
	50/M	Floor of mouth	N/A	ASCC	N/A	N/A
^11^	86/M	Gingiva	Nodular Tumor	ASCC	N/A	N/A
^12^	80/F	Posterior region of lower jaw	Swelling around body of mandible	ASCC	2 m	DOD 4 m
^13^	61/M	Tongue	Ulcerated nodular lesion	ASCC	2 m	DOD at 8 m
^14^	50/F	Gingiva	Ulcerated lesion	ASCC	18 m	DOD at 38 m
	56/M	Tongue	Erosive lesion	ASCC	26 m	DOD at 46 m
^15^	58/M	Gingiva	Verrucous nodular lesion	ASCC	No	NED 8 m
^16^	64/F	Floor of mouth	Erosion, red mass	ASCC	No	NED 5 m
^17^	56/M	Tongue	Ulcerated tumor	ASCC	5 m	DOD at 9 m
^18^	72/F	Gingiva (maxillary premolar)	Ulcerated tumor	ASCC	10 m	DOD at 7 m
^19^	70/F	Gingiva (mandible)	N/A	ASCC	N/A	N/A
^20^	38/F	Buccal mucosa	Ulcerated tumor	ASCC	3 m	DOD at 7 m
^21^	45/M	Floor of mouth	Erythematous swelling	ASCC	6 m	NED (6 from RT)
	53/M	Gingiva (anterior maxilla)	Ulcerated lesion	ASCC	N/A	N/A
^22^	68/F	Gingiva (anterior maxilla)	Exophytic polypoid mass	ASCC	1 m	DOD at 3 m
^23^	50/M	Gingiva (maxilla)	Ulcerated tumor	ASCC	N/A	N/A
^24^	55/M	Soft palate	Ulcerative lesion	ASCC	60 m	NED
^25^	76/F	Posterior mandibular region	Swelling on mandible	ASCC	N/A	N/A
^26^	59/F	Gingiva (maxillary premolar and palate)	Ulcerated verrucous lesion	ASCC	2 m	DOD at 8 m
^27^	70/F	Maxillary posterior region	Swelling from mid-palatine raphe till residual alveolar ridge	ASCC	N/A	N/A
^28^	59/F	Left posterior mandible	Pain and swelling	Clear cell SCC	N/A	N/A
^29^	52/M	Left side cheek	Ulceroproliferative lesion	Clear cell SCC	3 m	Disseminated disease
^30^	35/F	Left lateral border of tongue	Non healing ulcer for one month	Clear cell SCC	N/A	N/A
^31^	55/M	Posterior region of maxilla	Ulcerated swelling from 23 to 28	Clear cell SCC	No	5 m
^32^	66/F	Left lateral tongue	Fungating ulcerative lesion	Glycogen rich clear cell SCC	Lung metastasis	3 m
^33^	42/F	Lower jaw	Erythematous irregular white patches on the left alveolar mucosa	Clear cell SCC	N/A	N/A
^34^	60/M	Left posterolateral border of tongue	Ulcer on tongue	Clear cell SCC	N/A	Lost follow up
^35^	65/M	Left mandible	Ulcer on floor of mouth	Clear cell SCC	Recurrence to the tongue	metastasis to lung
^36^	70/M	Ventral surface of tongue	Exophytic mass	Clear cell SCC	N/A	N/A
index case	29/M	Right pterygomandibular raphe	Ulceroproliferative lesion	ASCC with clear cell change	No	2 m

ASCC = acantholytic squamous cell carcinoma; Clear cell SCC = clear cell squamous cell carcinoma; DOD = died of disease; F = female; M = male; m = month; N/A = not available; NED = no evidence of disease; RT = radiotherapy.

Demographics revealed that the affected age group ranged from 42 to 86 years (mean age 56 years), with the index case displaying an exceptionally younger age group (29 years), making it a unique standalone presentation. The current case demonstrates an atypical appearance of ASCC with obvious clear cell change is also included in the tabulated cases. Moreover, the present case was an avid hukkah smoker. The major etiologic factors for oral malignancies and risk factors for atypical lesions are tobacco smoke and hookah consumption. According to studies, hookah tobacco smoke contains hazardous substances such as carbon monoxide, heavy metals, and carcinogenic chemicals that directly cause oral cancer.^11,37^ ASCC prefers the vermilion surface of the lip or vulva, as well as the oral cavity, tongue, and nasopharynx.^38,39^ In 1977, Goldman et al.^40^ reported the first instance of ASCC in the oral cavity. Acantholysis term was derived from the Greek words akantha - thorn and loosening, to describe the loss of coherence between epithelial cells caused by the disintegration of their intercellular bridges. Acantholysis can also be characterized as primary or secondary in terms of pathogenesis.^41,42^ Primary acantholysis is thought to be caused by direct injury to the intercellular compartment, which can be either immunologic or hereditary, as seen in pemphigus and its variants, as well as other disorders such as Hailey-Hailey disease (HHD). Secondary acantholysis, on the other hand, develops due to keratinocyte changes or damage caused by circumstances. The disintegration of intercellular components preceded by keratinocyte damage could cause ASCC. Although the process of acantholysis in vesiculobullous lesions has been thoroughly explored, however the mechanism of acantholysis in lesions such as ASCC remains unknown. No definitive etiology has been identified because of the scarcity of cases and investigations on intra-oral ASCCs. However, several distinct mechanisms, such as Bhogavaram et al.^43^ have highlighted trauma as a factor. According to O'Shea et al.,^12^ the lack of cellular adhesion in ASCC is caused mostly by desmosomal abnormalities, specifically desmogleins 1 and 2, desmoplakin, or both. Another newly proposed mechanism by Krishnan et al.^44^ is collective cell migration and resistance to anoikis. Human Papillomavirus (HPV) is considered to be another causative agent for cutaneous ASCC, but its role is yet to be established in oral cavity ASCCs. TP53 mutations produced by UV light-induced DNA damage, activating mutations in HRAS, and loss-of-function mutations in Notch receptors that regulate normal squamous cell development are possible reasons for malignancies.^13^ HPV may be involved in one pathway but not others, accounting for its involvement in only a small percentage of all SCCs. HPV may disrupt cellular DNA repair or apoptotic pathways, making cells more vulnerable to UV-induced damage. HPV may function as a co-carcinogen with other variables to increase the cutaneous ASCC development. Lastly, it was concluded that it acted as an onlooker and was not a factor in the pathogenesis of acantholytic SCC.^14^ The discovery of a connection between HPV and ASCC could have diagnostic and/or therapeutic consequences. Understanding the mechanism of oncogenesis can enable better therapeutic targeting. Acantholysis and the aggressive behavior of ASCCs have been linked to changes in the expression of molecules such as E-cadherin, which mediates cell-cell and cell-extracellular matrix adhesions. Acantholysis-induced malignant cell detachment is associated with distant metastasizing behavior.^12^ ASCC is diagnosed histologically by the presence of a basic cell of the keratinizing squamous cell type, an adenoid structure consisting of a spherical area with a defined wall, primarily of one cell thickness, and a lumen containing single or grouped dyskeratotic acantholytic cells.

ASCC has several synonyms based on microscopic appearances, including adenoid SCC, pseudo glandular SCC, SCC with gland-like features, angiosarcoma-like SCC, pseudo vascular adenoid SCC, and epithelioma dyskeratoticum segregans. Some writers believe the biological behavior of ASCC of the oral cavity to have a poor prognosis. In contrast, others consider it to be varied, which could be related to the small number of documented cases. ASCC has many histopathological differentials, such as conventional SCC, which lacks duct-like structures with central acantholysis, and adenosquamous carcinoma, which shows true glandular differentiation with positivity for mucin in special stains such as PAS, mucicarmine, and alcian blue, which was ruled out. However, adenocarcinoma and mucoepidermoid carcinoma can be confused with ASCC; the presence of mucinous material aids in distinguishing. PAS staining was negative in this case, ruling out a glandular origin. Ancillary approaches, such as IHC, revealed substantial diffuse positivity for p40 and decreased expression of E cadherin within these dysplastic islands with acantholytic cells, assisting us in reaching a conclusive diagnosis.^15^ A high Ki-67 proliferative index (65%) was observed in the present case. Ki-67 has been used to assess the aggressiveness of a tumor and forecast its survival and prognosis due to its high sensitivity and specificity in labeling cell proliferation in malignant tissues. Higher Ki-67 expression is an indicator of tumor cells with higher proliferation and locally invasive potential and, thus providing one of the best markers for the evaluation of the biological aggressiveness of tumors.^15^

The clear cell alterations are attributed to significant hydropic degeneration of neoplastic cells and intercellular fluid buildup rather than stromal component accumulation such as lipid, glycogen, or mucus. Clear cells are prevalent in advanced SCC instances, according to Corbalán-Vélez et al.,^16^ implying a secondary phenomenon or clonal development. The WHO has recognized clear-cell SCC as a distinct entity in other locations, such as the penis and skin but not in the head-and-neck region, and these variations in other locations are known to be aggressive. Although the WHO 2022 classification does not include clear-cell SCC of the head and neck, there is available literature describing their occurrence and nature, and forecasting their behavior.^9,10^ Most instances of OSCC with obvious cell alterations have a dismal prognosis. However, due to the rarity of its occurrence, determining the clinical behavior of OSCC with obvious cell alterations is difficult. As a result, additional research is needed to understand better the clinical behavior and prognosis of these clear cell alterations identified in OSCC. As a result, the status of these clear cell changes is still debatable, with further data needed to recognize it as a variation. PAS staining should be performed to forecast the reason for clearing and rule out adnexal and salivary gland-origin neoplasms.

To summarize, oral ASCC with clear cell change is an exceptionally unusual presentation. The acantholytic process could be related to decreased expression of both adherens junctions and tight junctions’ molecular components, and clear cell change indicates advanced stages of OSCC.^12^ A few authors explained a unique epithelial-mesenchymal transition plasticity that states that when E-cadherin levels are moderately reduced, it leads to loss of epithelial coherence while remaining high enough for collective cell migration leading to stromal infiltration and metastasis, which may have an impact on the clinical outcome of the patients and clear cell change appears to be more of degenerative change.^44^

## CONCLUSION

Until recently, no case in the literature has emphasized such an atypical presentation of an acantholytic variant of OSCC with obvious cell change. To the best of our knowledge, this is the first documented occurrence. Our experience with the present case demonstrated a more aggressive behavior due to a high Ki-67 index, anticipating a poorer prognosis in the oral cavity given the patient's young age. Because ASCC in the oral cavity is uncommon, the oral pathologist may face a diagnostic quandary due to a lack of understanding of the tumor's etiology and histological characteristics. Clinicians should consider vigorous, multidisciplinary treatment and close follow-up with multimodality images if an ASCC with obvious clear cell change is diagnosed.

## References

[1] Sharma G, Kamboj M, Narwal A, Bhardwaj R, Yadav P (2022). Cytotoxic role of chlorogenic acid on oral squamous cell carcinoma cell line. Indian J Otolaryngol Head Neck Surg.

[2] Sankaranarayanan R, Ramadas K, Amarasinghe H, Subramanian S, Johnson N, Gelband H, Jha P, Sankaranarayanan R, Horton S (2015). Cancer: disease control priorities.

[3] Pathak J, Swain N, Patel S, Poonja L (2014). Histopathological variants of oral squamous cell carcinoma-institutional case reports. J Oral Maxillofac Pathol.

[4] Perez-Ordoñez B, Beauchemin M, Jordan RC (2006). Molecular biology of squamous cell carcinoma of the head and neck. J Clin Pathol.

[5] LeBoit PE, Weedon D, Sarasain A (2006). Pathology and genetics of skin tumours. World Health Organization classification of tumours.

[6] Goldman RL, Klein HZ, Sung M (1977). Adenoid squamous cell carcinoma of the oral cavity: report of the first case arising in the tongue. Arch Otolaryngol.

[7] Driemel O, Müller-Richter UD, Hakim SG (2008). Oral acantholytic squamous cell carcinoma shares clinical and histological features with angiosarcoma. Head Face Med.

[8] Kuo T (1980). Clear cell carcinoma of the skin. A variant of the squamous cell carcinoma that simulates sebaceous carcinoma. Am J Surg Pathol.

[9] Premalatha BR, Rao RS, Patil S, Neethi H (2012). Clear cell tumours of the head and neck: an overview. World J Dent.

[10] Kakoti LM, Mahanta D, Sharma JD, Chowdhury Z (2018). Clear-cell squamous cell carcinoma: an uncommon variant of very common malignancy in the head and neck. Int J Oral Health Sci.

[11] Taghibakhsh M, Farhadi S, Babaee A, Sheikhi M (2019). The effect of hookah use on buccal mucosa: evaluation of repair index. Asian Pac J Cancer Prev.

[12] O’Shea C, Fitzpatrick JE, Koch PJ (2014). Desmosomal defects in acantholytic squamous cell carcinomas. J Cutan Pathol.

[13] Arron ST, Ruby JG, Dybbro E, Ganem D, Derisi JL (2011). Transcriptome sequencing demonstrates that human papillomavirus is not active in cutaneous squamous cell carcinoma. J Invest Dermatol.

[14] Ratushny V, Gober MD, Hick R, Ridky TW, Seykora JT (2012). From keratinocyte to cancer: the pathogenesis and modeling of cutaneous squamous cell carcinoma. J Clin Invest.

[15] Raut T, Keshwar S, Jaisani MR, Shrestha A (2021). Adenoid (acantholytic) squamous cell carcinoma of mandibular gingiva. Case Rep Dent.

[16] Corbalán-Vélez R, Ruiz-Macia JA, Brufau C, López-Lozano JM, Martínez-Barba E, Carapeto FJ (2009). Las células claras en el carcinoma espinocelular cutáneo. Actas Dermosifiliogr.

[17] Ishikawa S, Ishikawa H, Kato T (2014). Acantholytic squamous cell carcinoma of the maxillary gingiva: case report and literature review. J Oral Maxillofac Surg Med Pathol.

[18] Takagi M, Sakota Y, Takayama S, Ishikawa G (1977). Adenoid squamous cell carcinoma of the oral mucosa: report of two autopsy cases. Cancer.

[19] Jones AC, Freedman PD, Kerpel SM (1993). Oral adenoid squamous cell carcinoma: a report of three cases and review of the literature. J Oral Maxillofac Surg.

[20] Kusafuka K, Ebihara M, Ishiki H (2006). Primary adenoid squamous cell carcinoma of the oral cavity. Pathol Int.

[21] Kerawala CJ (2009). Acantholytic squamous cell carcinoma of the oral cavity: a more aggressive entity?. Br J Oral Maxillofac Surg.

[22] Papadopoulou E, Tosios KI, Nikitakis N, Papadogeorgakis N, Sklavounou-Andrikopoulou A (2010). Acantholytic squamous cell carcinoma of the gingiva: report of a case and review of the literature. Oral Surg Oral Med Oral Pathol Oral Radiol Endod.

[23] Prasad KK, Kaur S (2010). Acantholytic squamous cell carcinoma of the oral cavity: an uncommon histological variant of squamous cell carcinoma. Minerva Stomatol.

[24] Yeoh MS, Kim DD, Ghali GE (2012). Acantholytic squamous cell carcinoma of the buccal mucosa: report of a case. J Oral Maxillofac Surg.

[25] Nayak SD, Jose M, Sequeira J (2012). Oral adenoid/acantholytic squamous cell carcinoma: a report of two cases with review of literature. Kathmandu Univ Med J.

[26] Ozgursoy OB, Tulunay O, Muz SE, Aslan G, Kucuk B (2013). Acantholytic squamous cell carcinoma of the maxilla: unusual location and aggressive behavior of a rare histologic variant. Ear Nose Throat J.

[27] Mardi K, Singh N (2014). Acantholytic squamous cell carcinoma of the oral cavity: a rare entity. J Oral Maxillofac Pathol.

[28] Lin JS, Lin HP, Liu CJ (2016). Acantholytic squamous cell carcinoma of soft palate-case report and literature review. J Dent Sci.

[29] Kang JH, Seo YK, Lee SR, Oh SH, Choi YS, Hwang EH (2019). Characteristic imaging findings of acantholytic squamous cell carcinoma: a case report. Oral Radiol.

[30] Kim JE, Lee C, Oh KY, Huh KH (2020). A rare acantholytic variant of squamous cell carcinoma of the maxilla: a case report and literature review. Medicine.

[31] Kulkarni M, Sahana NS, Suresh T, Renuga S, Khatoon H, Verghese R (2022). Adenoid (acantholytic) squamous cellcarcinoma of the alveolar ridge: a rare case report. J Oral Med Oral Surg Oral Pathol Oral Radiol.

[32] Frazier JJ, Sacks H, Freedman PD (2012). Primary glycogen-rich clear cell squamous cell carcinoma of the mandibular gingiva. Oral Surg Oral Med Oral Pathol Oral Radiol.

[33] Nainani P, Singh HP, Paliwal A, Nagpal N (2014). A rare case report of clear cell variant of oral squamous cell carcinoma. J Clin Diagn Res.

[34] Kaliamoorthy S, Sethuraman V, Ramalingam SM, Arunkumar S (2015). A rare case of clear cell variant of oral squamous cell carcinoma. J Nat Sci Biol Med.

[35] Devi A, Kamboj M, Singh V, Singh S (2017). Clear-cell variant of squamous cell carcinoma in maxilla as primary lesion: a rare case. J Oral Maxillofac Pathol.

[36] Khoury ZH, Bugshan A, Lubek JE, Papadimitriou JC, Basile JR, Younis RH (2017). Glycogen-rich clear cell squamous cell carcinoma originating in the oral cavity. Head Neck Pathol.

[37] Kamboj M, Sharma G, Narwal A, Gill PS, Devi A, Yadav J (2023). Estimation of serum leptin, adiponectin, and malondialdehyde levels in tobacco-induced oral squamous cell carcinoma: ELISA-based study. South Asian J Cancer.

[38] Zaatari GS, Santoianni RA (1986). Adenoid squamous cell carcinoma of the nasopharynx and neck region. Arch Pathol Lab Med.

[39] Jacoway JR, Nelson JF, Boyers RC (1971). Adenoid squamous-cell carcinoma (adenocanthoma) of the oral labial mucosa: a clinicopathologic study of fifteen cases. Oral Surg Oral Med Oral Pathol.

[40] Goldman RL, Klein HZ, Sung M (1977). Adenoid squamous cell carcinoma of the oral cavity: report of the first case arising in the tongue. Arch Otolaryngol.

[41] Kalele K, Deshmukh R, Alsi A (2018). A sneak peep at adenoid/acantholytic variant of oral squamous cell carcinoma. Oral Surg Oral Med Oral Pathol Oral Radiol.

[42] Seshadri D, Kumaran MS, Kanwar AJ (2013). Acantholysis revisited: back to basics. Indian J Dermatol Venereol Leprol.

[43] Bhogavaram B, Sinha R, Rapolu K, Boggarapu BP (2020). Adenoid squamous cell carcinoma of upper lip, a rare variant of squamous cell carcinoma: a case report. J Family Med Prim Care.

[44] Krishnan RP, Ramani P, Pandiar D (2022). Plausible mechanisms in the pathobiology of acantholytic squamous cell carcinoma: an evidence based hypothesis. Med Hypotheses.

